# Residential greenness, asthma, and lung function among children at high risk of allergic sensitization: a prospective cohort study

**DOI:** 10.1186/s12940-022-00864-w

**Published:** 2022-05-12

**Authors:** Kim Hartley, Patrick H. Ryan, Gordon L. Gillespie, Joseph Perazzo, J. Michael Wright, Glenn E. Rice, Geoffrey H. Donovan, Rebecca Gernes, Gurjit K. Khurana Hershey, Grace LeMasters, Cole Brokamp

**Affiliations:** 1 Cincinnati Children’s Hospital Medical Center, 3333 Burnet Avenue, Cincinnati, OH 45229 USA; 2 College of Nursing, University of Cincinnati, 3110 Vine St, Cincinnati, OH 45219 USA; 3 College of Medicine, University of Cincinnati, 3230 Eden Ave, Cincinnati, OH 45267 USA; 4 Toxic Effects Assessment Branch (Cincinnati), Chemical and Pollutant Assessment Division, Center for Public Health and Environmental Assessment (CPHEA), Office of Research and Development, U.S. Environmental Protection Agency, 26 West M.L. King Drive, Cincinnati, OH 45268 USA; 5 USDA Forest Service, PNW Research Station, 1220 SW 3rd Ave, Portland, OR 97204 USA; 6 Association of Schools and Programs of Public Health (ASPPH), Environmental Health Research Participant, 2014-2016, 1900 M St NW #710, DC 20036 Washington, USA

**Keywords:** Pediatric, Child, Greenspace, Green space, Respiratory, Allergy, Environment, Atopy

## Abstract

**Background:**

While benefits of greenness to health have been reported, findings specific to child respiratory health are inconsistent.

**Methods:**

We utilized a prospective birth cohort followed from birth to age 7 years (*n* = 617). Residential surrounding greenness was quantified via Normalized Difference Vegetation Index (NDVI) within 200, 400, and 800 m distances from geocoded home addresses at birth, age 7 years, and across childhood. Respiratory health outcomes were assessed at age 7 years, including asthma and lung function [percent predicted forced expiratory volume in the first second (%FEV_1_), percent predicted forced vital capacity (%FVC), and percent predicted ratio of forced expiratory volume in the first second to forced vital capacity (%FEV_1_/FVC)]. We assessed associations using linear and logistic regression models adjusted for community deprivation, household income, and traffic-related air pollution. We tested for effect measure modification by atopic status.

**Results:**

We noted evidence of positive confounding as inverse associations were attenuated upon adjustment in the multivariable models. We found evidence of effect measure modification of NDVI and asthma within 400 m at age 7 years by atopic status (*p* = 0.04), whereby children sensitized to common allergens were more likely to develop asthma as exposure to greenness increased (OR = 1.3, 95% CI: 0.9, 2.0) versus children not sensitized to common allergens (OR = 0.8, 95% CI: 0.5, 1.2). We found consistently positive associations between NDVI and %FEV_1_ and %FVC which similarly evidenced positive confounding upon adjustment. In the adjusted regression models, NDVI at 7 years of age was associated with %FEV_1_ (200 m: β = 2.1, 95% CI: 0.1, 3.3; 400 m: β = 1.6, 95% CI: 0.3, 2.9) and %FVC (200 m: β = 1.8, 95% CI: 0.7, 3.0; 400 m: β = 1.6, 95% CI: 0.3, 2.8; 800 m: β = 1.5, 95% CI: 0.1, 2.8). Adjusted results for %FEV_1_/FVC were non-significant except exposure at birth in the 400 m buffer (β = 0.81, 95% CI: 0.1, 1.5). We found no evidence of effect measure modification of NDVI by atopic status for objective measures of lung function.

**Conclusion:**

Sensitivity to allergens may modify the effect of greenness on risk for asthma in children but greenness is likely beneficial for concurrent lung function regardless of allergic status.

**Supplementary Information:**

The online version contains supplementary material available at 10.1186/s12940-022-00864-w.

## Background

Greenness, generally defined as vegetation such as trees and grasses, has shown promise for supporting mental and physical health [1–3]. Because of the immense global burden of respiratory disease [4], there is particular interest in the possibility that greenness may support respiratory health. Proposed pathways for this benefit include reducing harm via the enhancement of air quality [5, 6], and the biodiversity hypothesis, which proposes that greenness is protective against allergic and inflammatory disease by augmenting the human microbiome and promoting immune balance [7, 8]. However, reported associations between greenness and respiratory health in children so far have been divergent. Greenness has been found to be both a protective factor [5, 9–13] and a risk factor [14–16] for child respiratory health outcomes, while some studies have found no associations [17–20] or mixed results [21–23]. Two recent systematic reviews reported that published literature on the relationship between greenness and child asthma remain heterogenous in exposure and outcome assessment, limiting synthesis of findings and preventing meta-analysis [24, 25]. Similarly, a systematic review of findings among 11 cohorts examining the relationship between greenness and allergic sensitization in children found conflicting results and heterogeneity that precluded meta-analysis [26]. Studies investigating the relationship between greenness and child lung function are even scarcer, with one study reporting no significant association [27], while two found greenness beneficial for child lung function [28, 29].

The purpose of this study was to investigate how greenness around the home throughout childhood affects asthma and lung function in children at age 7 years. This study builds on previous literature by assessing greenness longitudinally, estimating air pollution exposure at the individual level, and examining objective measures of lung function to address discrepancies in extant literature for discerning relationships between greenness and respiratory health in children.

## Methods

### Study population

This study used a prospective cohort design to examine greenness and respiratory health among participants of the Cincinnati Childhood Allergy and Air Pollution Study (CCAAPS), a birth cohort of children recruited only if they lived far (> 1,500 m) or near (< 400 m) a major highway or interstate and had at least one parent who was sensitive to common aeroallergens [30, 31]. The study was conducted with approval of the relevant Institutional Review Boards, with parents providing written informed consent.

**Fig. 1:**
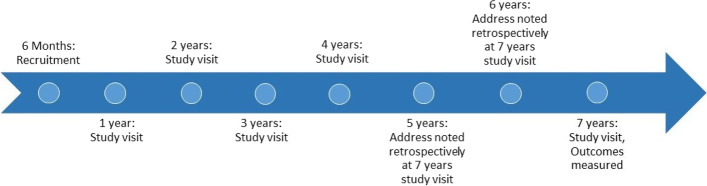
Timeline of participation in the Cincinnati Childhood Allergy and Air Pollution Study (CCAAPS) by age of participants

The CCAAPS cohort included 762 infants born between October 2001 and July 2003 in seven counties within southwest Ohio (Butler, Clermont, Hamilton, Warren) and northern Kentucky (Boone, Campbell, Kenton). Evaluations were conducted at ages 1, 2, 3, 4, and 7 years for various health and demographic characteristics including respiratory symptoms and residential addresses (Fig. 1). Although study visits were not conducted at ages 5 and 6 years, addresses for those time periods were collected retrospectively at the age 7 years visit [32]. Addresses were geocoded using EZLocate software from TeleAtlas as described by Ryan et al. [31].

### Greenness exposure assessment

Normalized Difference Vegetation Index (NDVI) was estimated from satellite measurements of visible and near-infrared light reflection from chlorophyll [33]. NDVI provides a continuous variable for greenness, with values ranging from -1 to + 1. Negative values are associated with water, near-zero values are associated with barrenness such as deserts, and positive values range from grassland to rainforests [33]. NDVI was extracted using Landsat Scene Path at 30 m resolution from cloud-free days in June in 2000 and 2010. For each study visit, the closest of the two NDVI scenes (2000 or 2010) to the time of data collection were used to calculate the average value within fixed distances (200, 400, and 800 m) from each participant’s geocoded residential address. Distances were chosen based on previous literature [15, 34, 35] and conceptualized to represent greenness immediately surrounding the home (200 m), at the street level (400 m), and in the surrounding neighborhood (800 m). This was estimated for three exposure time windows: birth, age 7 years, and averaged across childhood; the latter was calculated from the mean of NDVI values from each year between birth and age 7 years to provide a cumulative lifetime average.

### Respiratory outcome measurement

Measurements of respiratory outcomes including asthma and lung function in CCAAPS participants at age 7 years are described elsewhere [32]. Briefly, participants completed spirometry in accordance with American Thoracic Society criteria [36]. Lung function was measured as forced exhalation volume in the first second (FEV_1_) and forced vital capacity (reflecting the total volume of air exhaled; FVC). Percent predicted FEV_1_ (%FEV_1_) and percent predicted FVC (%FVC) were calculated by dividing individual values by the expected result for any person of similar age, sex, race, and height [37]. Ratio values of FEV_1_/FVC (reflecting the percentage of lung capacity expelled in one second) were similarly translated as percent predicted (%FEV_1_/FVC).

Participants suspicious for asthma (including those who had a %FEV_1_ equal to 90% or less, asthma symptoms in the previous 12 months, a reported physician diagnosis of asthma, or an exhaled nitric oxide level ≤ 20 ppb) received nebulized levalbuterol. Participants with less than 12% increase FEV_1_ upon repeat spirometry received a methacholine challenge test. As described by Reponen et al. [38], participants were defined as having asthma for the purposes of this study if they experienced symptoms of asthma during the study visit and demonstrated bronchodilation (≥ 1 2% increase FEV_1_ after nebulized levalbuterol) or bronchoprovocation (≥ 20% decrease baseline FEV_1_ after inhaled methacholine).

Participants were categorized as allergic or non-allergic based on skin-prick test results at the age 7 years study visit. As described by LeMasters et al. [30], participants were evaluated for sensitivity to cow’s milk, egg, and 15 aeroallergens including seven species of pollen, four species of mold, dog, cat, cockroach, and dust mite mix. Sensitivity was defined as a wheal ≥ 3 mm larger than the saline control after 15 min for at least one allergen [39].

### Covariate assessment

We used a causal inference approach to identify a minimally sufficient set of factors to adjust for confounding. We identified pathways confounding the estimation of the relationship between respiratory health outcomes and greenness a priori by constructing a directed acyclic graph (DAG; Supplemental Fig. 1) based on theorized causal relationships among variables [40]. Using DAGitty.net software [41], we identified household income (a marker of individual-level socioeconomic status), community deprivation (a neighborhood-level marker of socioeconomic status), and traffic-related air pollution as confounders to be adjusted for in our models.

#### Household income

Parent-reported household income was noted during age 1 year and age 7 years study visits. For analysis, income was categorized in values 1 through 9 and categories were treated as ordered factors (Table 1). Income at age 1 year was used in “Birth” models, income at age 7 years was used in “Age 7” models, and the two values were averaged for “Across Childhood” models.

**Table 1:** Characteristics, Health Outcomes, and Covariates by Age

	**Total Population** **n (%)**	**Asthmatic at Age 7** **n (%)**	**Not Asthmatic at Age 7** **n (%)**	***P*-value**^**#**^
**n (%)**	617 (100)	95 (16)^a^	494 (84)^a^	
**Sex**				0.18
Male	338 (55)	58 (61)	265 (54)	
Female	279 (45)	37 (39)	229 (46)	
**Race**				0.001
White	487 (79)	63 (66)	401 (81)	
Non-White	130 (21)	32 (34)	93 (19)	
**Household Income at Age 7**				< 0.001
Under $10 K	49 (8)	12 (13)	30 (6)	
$10 K to under $20 K	44 (7)	11 (12)	29 (6)	
$20 K to under $30 K	51 (8)	16 (17)	22 (4)	
$30 K to under $40 K	49 (8)	6 (6)	34 (7)	
$40 K to under $50 K	56 (9)	7 (7)	31 (6)	
$50 K to under $70 K	109 (18)	12 (13)	70 (14)	
$70 K to under $90 K	95 (15)	13 (14)	85 (17)	
$90 to under $110 K	76 (12)	4 (4)	78 (16)	
Over $110 K	63 (10)	13 (14)	109 (22)	
Did not respond	25 (4)	1 (1)	6 (1)	
**Allergic Sensitivity at Age 7**				
Yes	258 (42)	31 (33)	116 (23)	0.17
No	346 (56)	25 (26)	139 (28)	
Missing	13 (2)	39 (41)	239 (48)	
	**M (SD)**	**Asthmatic** **Mean**	**Non-Asthmatic Mean**	***P*-value**^**+**^
**ECAT (µg/m3)**				
Birth	0.40 (1.70)	0.42	0.40	0.08
Age 7 (missing = 6)	0.36 (0.11)	0.37	0.36	0.22
Across Childhood	0.38 (0.10)	0.40	0.38	0.06
**Community Deprivation Index**				
Birth (missing = 2)	0.46 (0.02)	0.52	0.45	< 0.001
Age 7	0.33 (0.14)	0.38	0.33	0.001
Across Childhood	0.40 (0.14)	0.46	0.39	< 0.001

*Abbreviations: ECAT* Elemental Carbon Attributable to Traffic, *CDI* Community Deprivation Index, *M* Mean, *m* Meter, *SD* Standard Deviation

^a^ Participants completing spirometry and airway bronchoconstriction, *n* = 589

^#^ *p*-value for Chi-square test; + *p*-value for t-test

#### Traffic-related air pollution

We estimated traffic-related air pollution as the fraction of elemental carbon attributable to traffic (ECAT) using a previously developed land use regression model based on ambient air sampling from 24 sites within the study area [42]. ECAT was estimated in “Birth” models using participant address at enrollment, estimates using home address at age 7 years were used in “Age 7” models, and estimates using home address for each year of life were averaged for “Across Childhood” models.

#### Community deprivation

We quantified community deprivation using a previously developed deprivation index [43]. Briefly, a principal component analysis of six census tract-level variables from the 2015 5-year American Community Survey related to material deprivation (fraction over age 25 with at least high school diploma or general educational development equivalent, fraction of households in poverty, median household income, fraction of population with no health insurance coverage, fraction of population receiving public assisted income or supplemental nutritional assistance, fraction of houses that are vacant) was used to create an index ranging from 0 to 1, with higher values denoting increasing deprivation. We estimated community deprivation index for three time windows: birth, age 7 years, and averaged across childhood. “Across Childhood” estimates were calculated from the mean of index values from each year between birth and age 7 years.

### Statistical analysis

We performed analyses using R [44]. We examined relationships between asthma status, measures of lung function, and NDVI in three buffer distances (200, 400, 800 m) at birth, age 7 years, and averaged across childhood using t-tests or Pearson correlation coefficients, as appropriate. We used logistic regression and linear regression modeling to estimate the effect of a 0.1-unit change in NDVI on asthma development and changes in lung function while adjusting for community deprivation index, household income, and ECAT. Participants with missing data for exposure or outcome constituted less than 5% of the population (< 1% NDVI, 4% lung function, 4.5% asthma) and were not included in the multivariate analyses.

Our “Birth” model included home address at birth, household income at age 1 year, ECAT at birth, community deprivation index at birth, and NDVI estimated from home address at birth. Our “Age 7” model included home address, household income, ECAT, community deprivation index, and NDVI estimated from home address at age 7 years. Our “Across Childhood” model included mean of household income at age 1 year and age 7 years, as well as mean of estimated annual ECAT, community deprivation index, and NDVI estimated from home addresses at each year of life.

To examine effect measure modification by allergic status, we used ANOVA to test for model fit improvement after addition of an interaction term between asthmatic status and NDVI. To limit problems with multiple testing for this exploratory test, only NDVI in the 400 m buffer distance at age 7 years was used, as this was the time and distance at which relationships were statistically significant. We considered an interaction to be present when *p* < 0.05.

## Results

Of 762 children enrolled in the CCAAPS cohort, 617 (81%) completed at least one feature of the age 7 years study visit (Table 1). Of these, 589 (95%) could be phenotyped based on completing spirometry with information on airway reversability/bronchoconstriction and were included in the analysis. Participants at age 7 years were similar to those who did not participate regarding sex and race, but differed in levels of maternal education and household income (Brunst et al., 2015). Fifty-five percent (*n* = 338) of participants were male, and the percentage of minority participants (*n* = 130, 21%) was similar to that of the sample region [45].

At age 7, asthma had been identified using physiologic criteria in 15% (*n* = 95) of participants. Mean %FEV_1_ and %FVC were nearly identical (µ = 1.02 for both, SD = 0.13 and 0.12 respectively, range 0.62–1.39) and similar to %FEV_1_/FVC (µ = 1.01, SD = 0.07, range 0.71–1.14; Table 1). No participants in the sample had %FEV_1_/FVC values that met the accepted threshold for clinical significance of less than 70% predicted [46].

There were no NDVI values in the raster or in our sample less than or equal to zero. Mean NDVI increased across all buffer distances between birth and age 7 years (200 m: 0.51 vs. 0.55, *p* < 0.001; 400 m: 0.53 vs. 0.58, *p* < 0.001; 800 m: 0.54 vs. 0.58, *p* < 0.001), indicating that participants were exposed to more greenness around the home as they aged (Table 2).

**Table 2:** Greenness exposure among 617 participants, as measured by NDVI, by time window and buffer radius

Time Window	NDVI		
	Buffer Distance	Mean (SD)	Range
Birth	200 m	0.51 (0.11)	0.10–0.77
	400 m	0.53 (0.10)	0.13–0.78
	800 m	0.54 (0.10)	0.15–0.74
Age 7	200 m	0.55 (0.09)	0.07–0.75
	400 m	0.58 (0.09)	0.13–0.77
	800 m	0.58 (0.09)	0.16–0.75
Across Childhood	200 m	0.53 (0.08)	0.18–0.72
	400 m	0.55 (0.08)	0.18–0.75
	800 m	0.56 (0.08)	0.17–0.74

*Abbreviations: m Meter, NDVI* Normalized Difference Vegetation Index, *SD* Standard Deviation

Average NDVI was higher for non-asthmatics than for asthmatics in “Birth” models for two buffer distances (200 m: 0.52 vs. 0.49, *p* = 0.04; 800 m: 0.54 v. 0.51, *p* = 0.01) and “Across Childhood” for one distance (200 m: 0.53 v. 0.51, *p* = 0.01) (Table 3). In multivariable models adjusted for confounding by community deprivation index, ECAT, and household income, NDVI was not statistically significantly associated with asthma for any distance in any time window (Table 3). We found the effect of NDVI on asthma within 400 m in “Age 7” models to be modified by allergic status (*p* = 0.04). Allergic participants may be more likely to develop asthma than non-allergic participants (allergic: OR = 1.3, 95% CI: 0.9, 2.0; non-allergic: OR = 0.8, 95% CI: 0.5, 1.2).

**Table 3:** Relationship between NDVI buffer distances and asthma across different time windows

Time Window	NDVI Buffer Distance	Mean NDVI			Adjusted
					Model Results^1^
		Non-Asthmatics	Asthmatics	*P*-value^2^	Odds Ratio (95% CI)^3^
Birth	200 m	0.52	0.49	0.04	0.99 (0.8, 1.2)
	400 m	0.53	0.51	0.07	0.99 (0.8, 1.3)
	800 m	0.54	0.51	0.01	0.84 (0.6, 1.1)
Age 7	200 m	0.56	0.54	0.09	0.94 (0.7, 1.2)
	400 m	0.58	0.57	0.23	1.01 (0.8, 1.3)
	800 m	0.58	0.57	0.21	1.01 (0.8, 1.4)
Across Childhood	200 m	0.53	0.51	0.01	0.97 (0.7, 1.3)
	400 m	0.55	0.54	0.16	1.13 (0.8, 1.6)
	800 m	0.56	0.54	0.08	1.04 (0.7, 1.5)

*Abbreviations: ECAT* Elemental Carbon Attributable to Traffic, *m* Meter, *NDVI* Normalized Difference Vegetation Index, *CI* Confidence Interval

^1^Logistic regression models represent the main effects adjusted for ECAT, household income, and community deprivation index

^2^*P*-value for t-test for difference of means

^3^Odds ratio per 0.1-unit change in NDVI

NDVI was positively correlated with %FEV_1_ and %FVC for all distances and time windows. No significant correlations between NDVI and %FEV_1_/FVC were found for any time window for any distance (Supplemental Table 1).

**Fig. 2:**
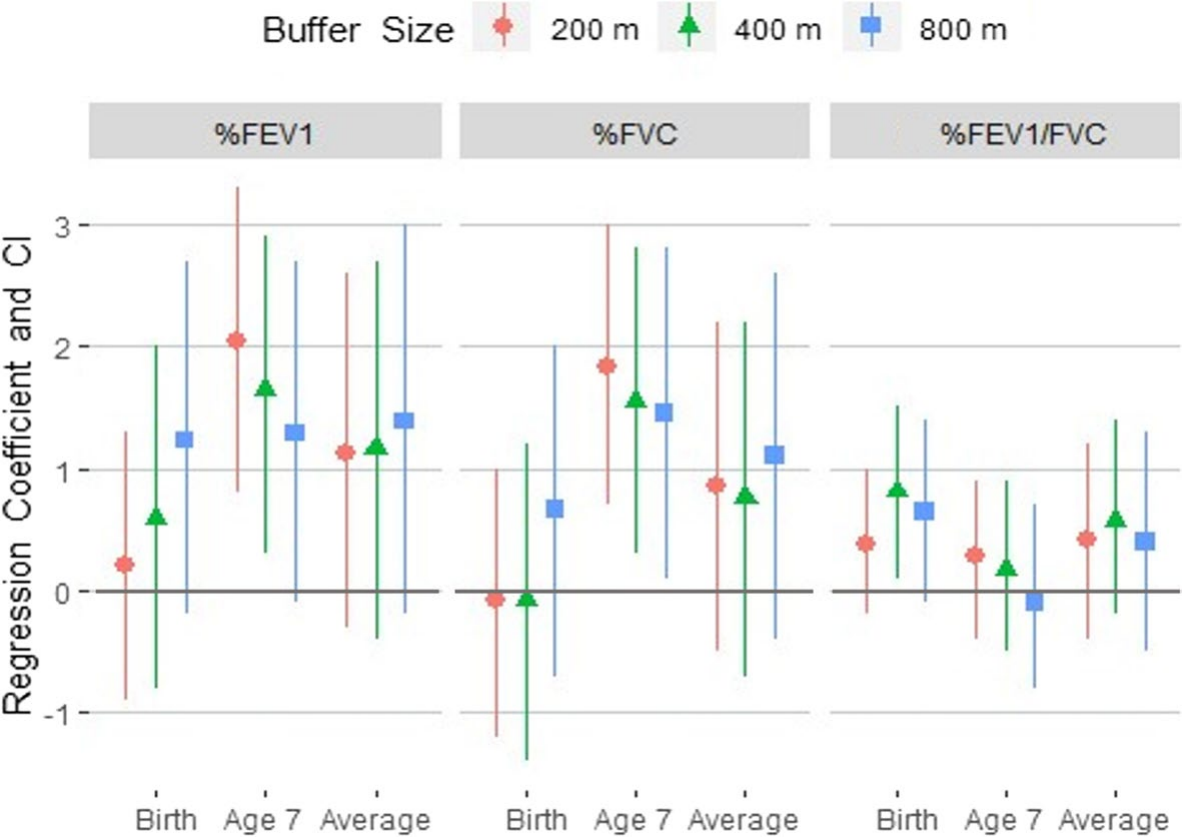
Plot of regression coefficients (β) and 95% confidence intervals for lung function after adjustment. Note. Models were adjusted for household income, elemental carbon attributable to traffic, and community deprivation index by buffer distance at birth, age 7, and averaged across childhood

While controlling for community deprivation index, ECAT, and household income, increased NDVI was significantly associated with increased %FEV_1_ at age 7 years for two distances (200 m: β = 2.1, 95% CI: 0.8, 3.3; 400 m: β = 1.6, 95% CI: 0.3, 2.9), but not in “Birth” or “Across Childhood” models for any distances (Fig. 2). A statistically significant increase in %FVC was found in all distances in “Age 7” models (200 m: β = 1.8, 95% CI: 0.7, 3.0; 400 m: β = 1.6, 95% CI: 0.3, 2.8; 800 m: β = 1.5, 95% CI: 0.1, 2.8), but not in “Birth” or “Across Childhood” models for any distances. For the %FEV_1_/FVC ratio, we only found one statistically significant association in the “Birth” model in the 400 m buffer (β = 0.8, 95% CI: 0.1, 1.5). We did not detect evidence of effect measure modification for any measure of lung function when examining groups by allergic status (%FEV_1_ *p* = 0.53, %FVC *p* = 0.74, %FEV_1_/FVC *p* = 0.71).

## Discussion

There is no current consensus regarding the effect of greenness on child asthma, as six studies have found protective associations [5, 9–13], three found adverse associations [14–16], three found no relationship [17–19], and three found mixed results within a single study [21–23]. Similar heterogeneity exists among published studies of greenness and child lung function, as one study found no relationship [27], and two found protective associations [28, 29]. However, to our knowledge this is the first study to examine the association between both asthma and clinically measured lung function in children and greenness at multiple distances using longitudinal exposure data. Consistent with recent studies [15, 17, 23] and a meta-analysis [47], we found no independent association between child asthma and surrounding greenness after adjusting for confounding. While Dzhambov et al. [10] found residential NDVI was marginally associated with lower prevalence of asthma among schoolchildren, stronger associations were noted in children without a family history of allergies. Their findings may differ from ours since our study population only included children with a family history of allergic sensitization. Further examination of the role of genetics within the relationship between greenness and asthma may be warranted. Additionally, our finding of some evidence of effect measure modification of greenness on asthma suggests that studies examining only main effects may be insufficient.

We found higher mean NDVI for non-asthmatics than asthmatics for all buffer distances and time windows. These unadjusted results were not evident in our multivariable regression analysis which indicates the effect of confounding. Selection bias cannot be completely ruled out, as our cohort experienced some attrition, with higher income and education levels for those participating at age 7 years than for those who originally enrolled in the study. Parents of asthmatic children may have also chosen to live in areas with less greenness. In a previous study, Brokamp et al. [48] reviewed residential mobility within the cohort, finding that over time, 54% of participants in this cohort moved at least once prior to age seven, with each move separated by a median of 4 miles and associated with a median decrease of 4% in traffic-related air pollution exposure, a 5% increase in greenspace, and an improved deprivation index.

Because approximately half of asthma cases are allergic in origin [49], there is concern that the introduction of pollen-producing vegetation may worsen asthma. However, according to the biodiversity hypothesis, early life exposures to a variety of microbial agents may be protective against development of allergic and inflammatory disease [7, 8]. Ecological analyses of the International Study of Asthma and Allergies in Childhood supported this hypothesis, suggesting that exposure to pollen may be protective for acquiring symptoms of respiratory disease [50]. Donovan et al. [9] similarly supported the hypothesis’ application to development of allergic asthma among children in New Zealand, finding vegetation diversity around a child’s home was protective for asthma. In their findings, it was specifically native vegetation that offered the best protection against development of asthma. This lends support to the biodiversity hypothesis as non-native vegetation reduces insect biomass, thereby decreasing child exposure to diverse organisms [51]. Building from the biodiversity hypothesis, city planning and greening initiatives could preserve existing native vegetation and increase native plantings to encourage biodiversity and potentially strengthen child immune function against allergic asthma and other immune-related diseases.

We found a 0.1-unit increase in NDVI was associated with an adjusted 1–2% increase in lung function (%FEV_1_ and %FVC) at age 7 years, with no discernable pattern for this benefit of greenness relative to buffer distances. Previous studies have shown an association between greenness and increased physical activity [52, 53]. Therefore, increased physical activity may be one pathway by which children in greener areas may exhibit higher %FEV_1_ and %FVC. This possibility is supported by a theorized mechanism that greenness contributes to human health through opportunities for physical activity [6]. While we were not able to examine this association as data on green space usage and activity tracking were not available, future studies should consider mediation analyses with these measures. While a 1–2% increase in lung function may seem minor on an individual level, this shift may have a larger impact for population health. Such benefits are evident when considered with findings that poor lung function at age 7 years is associated with adult chronic lung disease [54]. If these associations are causal, then it may be possible to lessen the burden of adult chronic lung disease by enhancing lung function during childhood through exposure to greenness.

Using longitudinal exposure data, we evaluated the potential etiologic window(s) for the observation of effects of exposure to greenness on child respiratory health. In consideration of the biodiversity hypothesis, we would perhaps expect that early-life exposures could manifest as positive associations between NDVI measured at birth and child lung function. However, we did not discern a benefit with increasing NDVI at birth, but we did find positive associations between NDVI at age 7 years and lung function. Given that age 7 years was the time our lung function tests were administered, it is possible that concurrent exposure to greenness confers benefit at any age. Administering lung function tests through additional years of follow-up may help further elucidate critical exposure windows in relation to lung function throughout childhood. A recent study using this approach to investigate the association between greenness exposure and lung function up to age 24 years found greenness in 100 m buffer distances from the home was positively associated with FEV_1_ and FVC [28]. However, exposure windows were not investigated. Interestingly, both our study and theirs found no differences between atopic and non-atopic individuals in relation to lung function.

We detected some consistency in adjusted models for NDVI and both %FEV_1_ and %FVC for nearly all buffer distances at age 7 years. These consistent results provide some reassurance that these findings were not likely spurious. We found only one statistically significant association between NDVI and %FEV_1_/FVC among both unadjusted and adjusted models. Lacking an evident pattern of association, this single finding may be spurious. Our pattern of null findings related to %FEV_1_/FVC are notable, since asthma is an obstructive disease characterized by a reduced FEV_1_/FVC ratio. Our finding is similar to a study by Lambert et al. [55] in which children exposed to pollen demonstrated similarly decreased FEV_1_ and FVC, but no effect was observed on FEV_1_/FVC.

A strength of our analysis included the availability of longitudinal data to examine multiple exposure windows. The use of stringent physiologic criteria to categorize asthma and allergic sensitivity reduced potential biases associated with self-report measures and is another strength of this analysis. While particulate matter (PM_2.5_), carbon monoxide (CO), nitrogen oxides (NO_2_), ozone (O_3_), and volatile organic compounds (VOCs) are commonly found in high concentrations in large cities, we chose to focus on a measure of elemental carbon attributable to traffic sources (ECAT), derived from ambient monitoring results of particulate matter with aerodynamic diameter < 2.5 μm [42]. Importantly, the recruitment of this birth cohort included proximity to major roadways; therefore, we believe there is a greater potential benefit of greenness on air pollution directly from local traffic sources in our mostly urban study area. Additionally, individually-derived ECAT estimates provide high spatial resolution that reduces exposure misclassification. Based on our inclusion criteria of children born to at least one atopic parent, the CCAAPS cohort is at high-risk for allergic sensitization. These collective study design features increased our overall enrollment of children who are genetically predisposed to developing allergic sensitivity. For example, we had a large sample size in the analyses stratified by allergic status, as nearly half of the participants were identified as atopic (*n* = 258, 42%). However, the odds ratios for both groups were somewhat imprecise, suggesting limited power to examine stratified results for effect sizes small in magnitude. The variation in greenness amongst participants enabled sufficient exposure contrasts to be examined, also strengthening our ability to detect associations.

Another strength of this study is our use of a DAG in identifying causal pathways between greenness and child respiratory health [56]. We theorized this causal pathway to include measures of individual-level socioeconomic status, community-level socioeconomic status, and air pollution. These were chosen based on previous evidence that household-level measures of socioeconomic status such as income may affect the development of respiratory diseases such as asthma [57]; traffic-related air pollution has been associated with increased asthma [58], atopy [59], allergic sensitization [60], and poor lung function [60]; and community-level indicators of socioeconomic status such as community deprivation level may affect respiratory illness [61]. Our DAG included urbanicity, which may serve as a proxy for other exposures such as air pollution and material community deprivation; however, our identified adjustment set without a direct measure of urbanicity was sufficient to calculate the direct effect of greenness on respiratory outcomes. While we have attempted to minimize confounding, as with all observational studies, the potential effect of unmeasured confounding could be a study limitation.

Among the CCAAPS cohort, NDVI around birth address at 400 m was highly correlated (*r* ≥ 0.99) for images in 2000 and 2010, indicating no major temporal changes to NDVI over those ten years. Consequently, we expect any exposure measurement error from longer-term temporal variability that may exist to be non-differential. Further, while we recognize increasing interest in the effect of blue spaces [62], we were unable to examine these associations as neither our NDVI raster nor our dataset included negative NDVI values that would indicate proximity to water. Although we estimated greenness by measuring NDVI because it provides an objective measure of chlorophyll, a limitation of the measure is that it does not differentiate species of vegetation. This could be important for examining hypothesized relationships between child respiratory disease and exposure to aeroallergens such as pollen [34] or for detecting effect measure modification by atopy. For example, two recent studies have found grass pollen [63] and evergreen pollen [55] negatively associated with lung function in children and adolescents. Markevych et al. [22] found that both asthma and allergic sensitivity increased as exposure to trees, and specifically allergenic trees, increased. Those results and our findings here highlight the potential importance of considering species among greenness when examining respiratory outcomes in future research. Further, NDVI does not discern the quality of greenness nor does it provide information regarding children’s patterns of use of green spaces, and our estimates of NDVI did not consider seasonal variation. It has also been noted that NDVI may suffer from pixel contamination [64]. While these limitations may increase the potential for exposure measurement error, we believe that the consistency and complete geographic coverage of this estimated exposure over our entire study area outweighs the benefits of other estimates of exposure that may have slightly better accuracy. Importantly, previous research has found NDVI to be an acceptable measure of greenness in epidemiological studies within urban areas like ours, especially in buffer sizes over 100 m [65]. A notable advantage of NDVI is that it continues to be commonly used in analyses of greenness because it can be freely downloaded for any geographic area of the world, providing consistency in measurement that enables replication. Such consistency in greenspace exposure measures used across studies may aid synthesis of findings and meta-analysis, which is important for drawing causal inferences and potential intervention opportunities based on the relationships examined here. Researchers can meet both of these needs, advance exposure assessment in the field, and potentially enable determination of specific mechanisms by which greenness is associated with health through use of NDVI together with other measures of greenness in future studies, such as tree canopy cover, street tree count, and more comprehensive novel indices [10, 34].

## Conclusions

Based on our findings, residential greenness appears protective against asthma development in children who are not sensitive to common allergens, but greenness can negatively affect asthmatic status in children who are allergic. Early and continued exposure to greenness may be most supportive of lung function in children regardless of allergic sensitivity, as current exposure was most beneficial for lung function in the study population, with no significant difference in benefit between children who were allergic and non-allergic. Future studies should consider allergic sensitivity when investigating the relationship between asthma and greenness and consider measures of greenness that can differentiate species of vegetation to build a body of evidence regarding the complex relationship between greenness and child respiratory health.

## Supplementary Information

**Additional file 1: Supplemental Table 1.** Analysis of Lung Function (percent predicted FEV_1_, percent predicted FVC, percent predicted FEV_1_/FVC) and NDVI.

**Additional file 2: Supplemental Figure 1. **Directed acyclic graph (DAG) of child respiratory health and greenness.

## Data Availability

The datasets generated and/or analyzed during the current study are not publicly available but are available from the corresponding author upon reasonable request.

## Abbreviations

CCAAPS: Cincinnati Childhood Allergy & Air Pollution Study
ECAT: Elemental carbon attributable to traffic
FEV_1_: Forced expiratory volume in the first second
FEV_1_/FVC: Ratio of forced expiratory volume in the first second / forced vital capacity
FVC: Forced vital capacity
NDVI: Normalized Difference Vegetation Index
