# Supplementary material for: Residential greenness, asthma, and lung function among children at high risk of allergic sensitization: a prospective cohort study
Source: Environ Health. 2022 May 12;21:52. doi: 10.1186/s12940-022-00864-w (PMC9097404; doi:10.1186/s12940-022-00864-w)
Supplement: Supplementary file 1 — Additional file 1: Supplemental Table 1. Analysis of Lung Function (percent predicted FEV1, percent predicted FVC, percent predicted FEV1/FVC) and NDVI. [file 12940_2022_864_MOESM1_ESM.docx]

Supplemental Table 1.

*Analysis of Lung Function (percent predicted FEV_1_, percent predicted FVC, percent predicted FEV_1_/FVC) and NDVI.*

|  |  | % Predicted FEV_1_ | | | % Predicted FVC | | | % Predicted FEV_1_/FVC | | |
| --- | --- | --- | --- | --- | --- | --- | --- | --- | --- | --- |
| Age | NDVI Buffer | Pearson Correlation | P-value | β (CI) | Pearson Correlation | P-value | β (CI) | Pearson Correlation | P-value | β (CI) |
| Birth | 200m | 0.05 | 0.24 | 0.20 (-0.9, 1.3) | 0.04 | 0.28 | -0.08 (-1.2, 1.0) | 0.02 | 0.67 | 0.38 (-0.2, 1.0) |
|  | 400m | 0.06 | 0.14 | 0.59 (-0.8, 2.0) | 0.05 | 0.27 | -0.08 (-1.4, 1.2) | 0.04 | 0.29 | 0.81 (0.1, 1.5) |
|  | 800m | 0.09 | 0.02 | 1.23 (-0.2, 2.7) | 0.09 | 0.03 | 0.67 (-0.7, 2.0) | 0.02 | 0.58 | 0.65 (-0.1, 1.4) |
| Age 7 | 200m | 0.17 | 0.00 | 2.05 (0.8, 3.3) | 0.17 | 0.00 | 1.84 (0.7, 3.0) | 0.02 | 0.70 | 0.28 (-0.4, 0.9) |
|  | 400m | 0.14 | 0.00 | 1.64 (0.3, 2.9) | 0.15 | 0.00 | 1.55 (0.3, 2.8) | 0.01 | 0.90 | 0.17 (-0.5, 0.9) |
|  | 800m | 0.12 | 0.00 | 1.29 (-0.1, 2.7) | 0.14 | 0.00 | 1.45 (0.1, 2.8) | -0.01 | 0.75 | -0.10 (-0.8, 0.7) |
| Across Childhood | 200m | 0.10 | 0.01 | 1.12 (-0.3, 2.6) | 0.10 | 0.01 | 0.86 (-0.5, 2.2) | 0.01 | 0.50 | 0.41 (-0.4, 1.2) |
|  | 400m | 0.10 | 0.02 | 1.17 (-0.4, 2.7) | 0.09 | 0.02 | 0.76 (-0.7, 2.2) | 0.03 | 0.50 | 0.57 (-0.2, 1.4) |
|  | 800m | 0.11 | 0.01 | 1.38 (-0.2, 3.0) | 0.11 | 0.01 | 1.11 (-0.4, 2.6) | 0.01 | 0.82 | 0.40 (-0.5, 1.3) |
| β = Change in percent predicted of measured outcome (FEV_1_, FVC, or FEV_1_/FVC) per 0.1 change in NDVI; CI = 95% Confidence Interval | | | | | | | | | | |
| Adjusted for ECAT, household income, and community deprivation index | | | | | |  |  |  |  |  |
