# Supplementary material for: Residential greenness, asthma, and lung function among children at high risk of allergic sensitization: a prospective cohort study
Source: Environ Health. 2022 May 12;21:52. doi: 10.1186/s12940-022-00864-w (PMC9097404; doi:10.1186/s12940-022-00864-w)
Supplement: Supplementary file 2 — Additional file 2: Supplemental Figure 1. Directed acyclic graph (DAG) of child respiratory health and greenness. [file 12940_2022_864_MOESM2_ESM.docx]

**Supplemental Figure 1**

*Directed acyclic graph (DAG) of child respiratory health and greenness.*


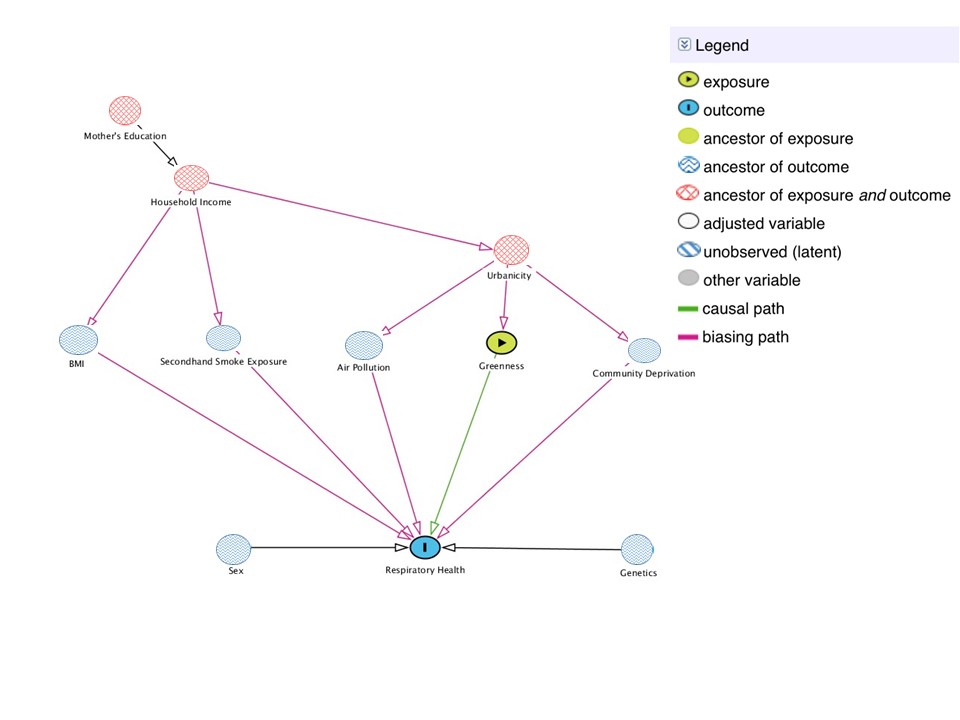
*Note.* This DAG was used to identify confounding pathways and choose model covariates. Created using DAGitty.net software (Textor et al., 2016).
